# A Randomized Controlled Trial of a Digital Stepped‐Care Program to Reduce Anxiety and Depressive Symptoms Among Long‐Term Care Workers With Psychological Distress: The REPICAL Study

**DOI:** 10.1155/da/5875889

**Published:** 2026-10-02

**Authors:** Leticia González-Spinoglio, Mireia Esplugues-Tormo, Iago Giné-Vázquez, Jordi Rodeiro-Boliart, Salvatore Aguilar-Ortiz, Jose Luis Ayuso-Mateos, Kerry R. McGreevy, Roberto Mediavilla, Rinske Roos, Marit Sijbrandij, Anke B. Witteveen, Josep Maria Haro, Mireia Felez-Nobrega

**Affiliations:** ^1^ Institut de Recerca Sant Joan de Déu (IRSJD) Parc Sanitari Sant Joan de Déu Sant Boi de Llobregat (Barcelona) Spain pssjd.org; ^2^ Departament de Medicina Facultat de Medicina i Ciències de la Salut Universitat de Barcelona (UB) Barcelona Spain ub.edu; ^3^ Centro de Investigación Biomédica en Red de Salud Mental (CIBERSAM) Instituto de Salud Carlos III Madrid Spain isciii.es; ^4^ Department of Psychiatry Universidad Autónoma de Madrid Madrid Spain uam.es; ^5^ Department of Clinical Neuro- and Developmental Psychology WHO Collaborating Center for Research and Dissemination of Psychological Interventions Amsterdam Public Health Institute Vrije Universiteit Amsterdam Amsterdam Netherlands vu.nl

**Keywords:** depression, digital mental health interventions, eHealth, health-related quality of life, home-care workers, mental health, post-traumatic stress disorder, scalable interventions, telemedicine

## Abstract

**Background:**

Long‐term care workers (LTCWs), encompassing those in long‐term care facilities and home‐care settings, constitute a particularly vulnerable population to psychological distress. This study aimed to evaluate the effectiveness of a digital stepped‐care program, adapted from two World Health Organization (WHO) psychological interventions—Doing What Matters in Times of Stress (DWM) and Problem Management Plus (PM+)—in reducing anxiety and depressive symptoms among LTCWs.

**Methods:**

A parallel‐group randomized controlled trial was conducted. Participants were LTCWs from Catalonia (Spain) experiencing psychological distress (Kessler Psychological Distress Scale, K10 ≥ 16). All participants received Psychological First Aid (PFA), and those in the intervention group subsequently received the digital stepped‐care program: DWM as step 1, and if psychological distress persisted (K10 ≥ 16), PM + as step 2. The primary outcome was the between‐group difference in self‐reported depressive and anxiety symptoms at week 21 (2‐month post‐program follow‐up), measured using the Patient Health Questionnaire–Anxiety and Depression Scale (PHQ‐ADS). Secondary outcomes included depressive and anxiety symptoms measured separately, post‐traumatic stress disorder (PTSD) symptoms, and health‐related quality of life (HR‐QoL; measured using PHQ‐9, 7‐item Generalized Anxiety Disorder [GAD‐7] scale, PCL‐5, and EuroQol 5‐dimensional descriptive system–5‐level [EQ‐5D‐5L], respectively).

**Results:**

Between October 2022 and May 2024, 165 participants were randomized (intervention: *n* = 83; control: *n* = 82). At week 21, depressive and anxiety symptoms were significantly lower in the intervention group, with moderate to large effect sizes (mean difference = 4.2, 95% CI: 1.1–7.3; Standardized effect size [SES] = 0.8, 95% CI: 0.1–1.4). Secondary outcomes also showed significantly greater improvement in the intervention group, except for HR‐QoL, for which no differences were found.

**Conclusions:**

This study provides evidence supporting the effectiveness of a digital and scalable stepped‐care program to support LTCWs’ mental health. These robust findings may inform occupational and public health strategies for supporting LTCWs’ experiencing psychological distress.

**Trial Registration:** ClinicalTrials.gov identifier: NCT05526235

## 1. Introduction

Mental health problems are highly prevalent in the working population, accounting for around 40% of reported cases of occupational diseases [1, 2]. Long‐term care workers (LTCWs), encompassing those in long‐term care facilities and home‐care settings, constitute a particularly vulnerable population for mental health problems. This group, characterized by a predominantly female workforce, faces considerable work‐related stress due to the physically and emotionally demanding nature of their tasks [3]. Despite their crucial role in providing essential care to vulnerable individuals, LTCWs face precarious working conditions, including heavy workloads, rotating shifts, limited training and resources, inadequate pay, and a lack of social and professional recognition [4, 5]. All these factors have been consistently identified as work‐related risk factors for mental health problems [6, 7]. Furthermore, the COVID‐19 pandemic exacerbated this scenario [4, 8], with nearly 60% of the workforce experiencing clinically relevant levels of depression, anxiety, and/or post‐traumatic stress disorder (PTSD) symptoms following the outbreak [9, 10], and with the mental health burden persisting over time [11].

Poor mental health among LTCWs may extend beyond individual well‐being to potentially jeopardize the quality of care and patient safety [12]. Moreover, it leads to increased rates of presenteeism, absenteeism, and employee turnover, impacting workers, patients, employers, and the broader economy [13].

Therefore, implementing strategies to improve the mental health in this population group is imperative. Nevertheless, psychological interventions targeting LTCWs’ mental health remain scarce. A systematic review by Herz et al. [14] analyzed 23 randomized controlled trials on health promotion strategies for this workforce over the past two decades, yet only four focused on psychological interventions. Mindfulness‐based [15, 16], acceptance and commitment therapy (ACT)‐based [17], and positive psychology [18] interventions showed some promise in reducing psychological distress, burnout, and compassion fatigue, as well as enhancing job satisfaction. However, their effectiveness was inconsistent; only one study directly assessed anxiety symptoms and found no significant effect, and none directly assessed depressive symptoms.

In this context, the current study aimed to evaluate the effectiveness of a digital stepped‐care program, adapted from two World Health Organization (WHO) psychological interventions—Doing What Matters in Times of Stress (DWM) and Problem Management Plus (PM+)—in reducing anxiety and depressive symptoms among LTCWs experiencing psychological distress. To achieve this, we (1) adapted these WHO interventions to be delivered digitally and to suit LTCWs needs and (2) conducted a randomized controlled trial to assess the stepped‐care program’s effectiveness.

Our primary hypothesis was that, at the final follow‐up (2 months after PM+), LTCWs allocated to the digital stepped‐care program (intervention group) would present lower levels of depressive and anxiety symptoms than those in the control group. Secondary hypotheses were that, at the final follow‐up, participants in the intervention group would present lower levels of PTSD symptoms and higher health‐related quality of life (HR‐QoL) compared with those in the control group.

## 2. Methods

### 2.1. Adaptation

Our stepped‐care program is an adaptation of two WHO psychological interventions, Doing What Matters in Times of Stress: An Illustrated Guide [19], part of the WHO stress management program Self‐Help Plus (SH+) [20] and PM+: individual psychological help for adults impaired by distress in communities exposed to adversity [21]. DWM is a stress management intervention based on ACT, which aims to reduce stress and increase resilience [19]. PM+ is an intervention designed for common mental health problems (e.g., depression, anxiety, and posttraumatic stress symptoms), based on cognitive–behavioral therapy (CBT), aimed at providing effective tools for problem management [21]. These interventions are widely applicable to diverse mental health problems and easily adaptable to various populations, cultures, and languages. They are open access and can be delivered through multiple modalities, including digital tools [22, 23]. In the adaptation process, we focused on enabling digital delivery and tailoring the program to suit the specific needs of our target population.

We began by performing a qualitative study to identify LTCWs’ psychological needs and to explore barriers and facilitators related to the use of digital mental health tools [11]. The information obtained from this analysis guided the adaptation of the interventions. Following this, and building on a previously adapted version used in the RESPOND study [24], we proceeded with the translation, cultural adaptation, and digital formatting of the interventions. The original DWM guide was transformed into a user‐friendly web and mobile application (available for Android and iOS devices). The written content was translated to Catalan (the Spanish version was already in place), and audios were recorded in Catalan and re‐recorded in European Spanish to ensure that the entire platform is available in both local languages. In comparison to the original manual, the number of images was reduced to simplify and create a more fluent and dynamic navigation experience. The images included were chosen based on their relevance, with a focus on diversity (representing different ethnicities, sexual orientations, and ages) and a commitment to gender equality. Additionally, some images were newly created to accurately reflect the context and population of the study. PM+ was adapted to be delivered using teleconference tools. The session’s duration was shortened from 90 to 60 min to fit the delivery mode better. In terms of content, the use of case examples was also tailored to facilitate relatability. Lastly, the result of the content adaptation was reviewed by two psychologists to assess its accuracy and comprehensibility. Overall, once the digitization was completed, multiple user experience tests were performed with the DWM web/app and the videoconference platform Jitsi Meet, used for PM+ video conferences.

### 2.2. Randomized Controlled Trial

#### 2.2.1. Study Design

The REPICAL study was a two‐arm, parallel‐group, randomized controlled trial (allocation ratio 1:1), with the data analyst blinded to treatment allocation. The participants were LTCWs from the community of Catalonia (Spain). All participants received Psychological First Aid (PFA), but only the intervention group received the digital stepped‐care program (see Interventions). The program was delivered remotely by trained junior mental health professionals and supervised by a senior clinical psychiatrist. All participants self‐reported measures at the following time points: T1: baseline; T2: week 7 (1 week after DWM); T3: week 13 (1 week after PM+); and T4: week 21 (2 months after PM+). The study was approved by the Ethics Committee of Parc Sanitari Sant Joan de Déu (Barcelona), and all participants provided written informed consent. Recruitment took place from October 2022 to May 2024. This report was written following the Consolidated Standards of Reporting Social and Psychological Intervention Trials (CONSORT‐SPI) guidelines [25].

#### 2.2.2. Participants

##### 2.2.2.1. Recruitment

Several strategies were employed. We collaborated with various stakeholders, including nursing home organizations, home caregiver associations, and labor unions, reaching LTCWs through different communication channels (internal communications, workplace posters, and meetings). We also disseminated the study through social media campaigns on Instagram and Facebook. Interested individuals contacted the research team via a form on the project’s website.

##### 2.2.2.2. Screening

The screening was conducted via a phone call, with data collected using the REDCap electronic data capture software. The following inclusion/exclusion criteria were applied:

Inclusion criteria:

– Being 18 years old or older.
– Being a worker in a long‐term care facility or a home‐care worker in the community of Catalonia.
– Experiencing psychological distress: Kessler Psychological Distress Scale (K10) ≥ 16.
– Being able to read and speak Catalan and/or Spanish.

Exclusion criteria:

– Currently receiving formal mental health care at the time of screening (provided in specialized services or primary care centers).
– Initiation, cessation, or substantial modification of a psychopharmacological treatment within the previous 2 months.
– Clinical evidence of a high or imminent suicide risk.
– Clinical evidence of compromised decision‐making capacity.

To evaluate psychological distress, we applied the Kessler Psychological Distress Scale (K10) [26]. The K10 is a self‐reported questionnaire that measures nonspecific psychological distress experienced in the past month based on questions about anxiety and depressive symptoms. It consists of 10 items that can be ranked from 1 (“none of the time”) to 5 (“all of the time”). Final score ranges from 10 to 50 and is obtained by summing all the items. Higher scores represent higher levels of distress. In line with previous research and established K10 cut‐offs, a score of 16 or higher, reflecting at least moderate psychological distress, was required for study entry [27–31]. This criterion was used to define a clinically meaningful starting point for study interventions. Suicidal ideation was assessed using the PM+ manual suicidal thoughts interview [21]. In cases where suicide risk was detected, a dedicated action protocol was followed. No withdrawal criteria were established. Participants had the right to withdraw from the study at any point.

##### 2.2.2.3. Group Allocation

Assignment of participants to the intervention or control group was conducted by the project coordinator using REDCap, following a random allocation sequence with blocks of unequal sizes (4 and 6) and a 1:1 allocation ratio. The project coordinator was unaware of the allocation sequence design, did not participate in the screening process, and did not deliver any intervention as an emotional management facilitator. Neither participants nor emotional management facilitators were blinded to allocation due to the nature of the behavioral interventions.

#### 2.2.3. Interventions

All participants were allowed to continue their regular care. This included informal psychological support, stabilized psychopharmacological treatment, or other forms of support, such as community care, psychoeducation, or social and legal assistance.

##### 2.2.3.1. PFA

Participants received PFA at the beginning of the call in which they were informed of their group allocation (control or intervention), and it lasted 15–20 min. PFA is a WHO developed intervention that offers compassionate, supportive, and practical assistance to individuals recently exposed to significant stressors [32]. During this session, participants could freely share their concerns and receive attentive and comforting support. If deemed necessary, this support included information about available community and health resources.

##### 2.2.3.2. Stepped‐Care Program

Participants allocated to the intervention group received the stepped‐care program. Step 1 consists of DWM and step 2 consists of PM+. All participants in the intervention group received PFA and Step 1. Step 2 was offered only to those participants who still met the criteria for psychological distress (K10 ≥16) 1 week after completing step 1 (Figure 1).

**Figure 1:**
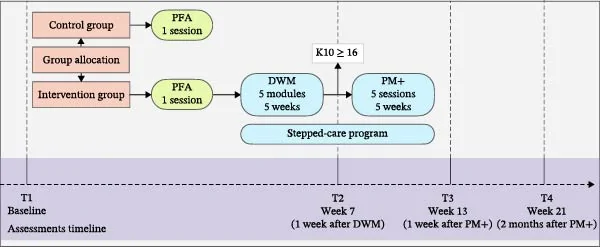
Interventions and assessments timeline. DWM, Doing What Matters in Times of Stress; K10, Kessler Psychological Distress Scale; PFA, Psychological First Aid; PM+: Problem Management Plus.

Step 1: DWM in times of stress. This intervention, accessed through the app/web, consists of five modules designed to enhance stress management using ACT‐based techniques. The modules are: “Grounding,” “Unhooking,” “Acting on your values,” “Being kind,” and “Making room.” Although this is a self‐guided intervention, each participant was assigned an emotional management facilitator (henceforth referred to as “helper”) who provided support through a 15 min weekly call, one per module, scheduled in advance. The intervention spans a total of 5 weeks. More details about the intervention are provided in the Supporting Information.

Step 2: PM+. This intervention consists of five 60 min individual videoconference sessions, each focused on a different CBT technique to develop effective problem‐management skills. The sessions are: “Managing stress,” “Managing problems,” “Get going, keep doing,” “Strengthening social support,” and “Staying well.” Conducted by the participant’s helper, sessions were held weekly over 5 weeks. More details about the intervention can be found in the Supporting Information.

Helpers providing support in the stepped‐care program were junior psychologists and psychiatrists. A total of seven helpers participated in the study (6 women and 1 man). They underwent a specific 4‐day training to conduct these interventions, facilitated by the clinical supervisor and project coordinators. Protocol adherence and clinical support for the helpers were ensured through weekly supervisions led by the clinical supervisor. The supervisor was a senior psychiatrist who had completed a 9‐day training‐of‐trainers program led by a WHO master trainer. Additionally, we conducted a treatment fidelity evaluation, which included (a) checklists completed by helpers after each session, as well as by the supervisor after each supervision and (b) evaluation of a randomly selected 10% of session audio recordings (only the helper was recorded) using fidelity checklists.

#### 2.2.4. Assessments

Quantitative assessments occurred at various time points: T0 (screening), T1 (baseline), T2 (week 7, 1 week after DWM), T3 (week 13, 1 week after PM+), and T4 (week 21, 2 months after PM+). Baseline (T1) and follow‐up assessments (T2, T3, and T4) were conducted using the online platform Qualtrics. Questionnaires were administered consistently across all assessments, with two exceptions: sociodemographic variables were exclusively collected at T1 and presented at the beginning of the assessment, while the screening instrument K10 was reevaluated only at T2 and was also presented at the beginning of the assessment. The estimated completion time for the assessments was 10–15 min. Figure 1 illustrates the assessment timeline. Participants who successfully completed at least three of the four assessments received a €20 voucher as a token of appreciation for their time and effort.

#### 2.2.5. Outcomes

##### 2.2.5.1. Primary Outcome

The primary outcome was the between‐group difference in depressive and anxiety symptoms at T4 (week 21), measured using the Patient Health Questionnaire–Anxiety and Depression Scale (PHQ‐ADS). This 16‐item self‐report scale combines the 9‐item PHQ (PHQ‐9), which assesses depressive symptoms, and the 7‐item Generalized Anxiety Disorder scale (GAD‐7), which assesses anxiety symptoms [33]. Respondents rate the frequency of each symptom over the past 2 weeks on a four‐point Likert scale ranging from 0 (“not at all”) to 3 (“nearly every day”). Total scores ranged from 0 to 48, with higher scores indicating greater symptom severity. The Spanish versions of PHQ‐9 and GAD‐7 have been validated [34, 35].

##### 2.2.5.2. Secondary Outcomes

Depressive Symptoms: PHQ‐9, the subscale of PHQ‐ADS that measures depressive symptoms during the past 2 weeks, was used. This scale asks about the nine DSM‐5 depression criteria and ranges from 0 to 27, with a higher score indicating a higher level of symptomatology [36].

Anxiety Symptoms: GAD‐7, the subscale of PHQ‐ADS that asses the degree of anxiety symptoms over the last 2 weeks, was used [37]. The total score ranges from 0 to 21, with a higher score indicating a higher level of symptomatology [35].

PTSD Symptoms: The shortened 8‐item version of the self‐reported 20‐item PTSD checklist for DSM‐5 (PCL‐5) was used [38, 39]. This instrument assesses PTSD symptoms based on the DSM‐5 diagnostic criteria. Respondents were asked about the extent to which each symptom had impacted them in the past month, providing responses from 0 (“not at all”) to 4 (“extremely”). The total score ranged from 0 to 32, with a higher score indicating a higher level of symptomatology.

HR‐QoL: This outcome was measured using the EuroQol 5‐dimensional descriptive system–5‐level version (EQ‐5D‐5L) [40]. The EQ‐5D‐5L assesses five dimensions: mobility, self‐care, usual activities, pain/discomfort, and anxiety/depression. Each dimension is rated on five levels of impairment: “none,” “slight,” “moderate,” “severe,” and “extreme” (coded 1–5, respectively). Higher EQ‐5D‐5L scores indicate a better HR‐QoL. Spanish country‐specific value sets were applied according to the EQ‐5D‐5L valuation protocol [41], based on the Spanish valuation study by Ramos‐Goñi et al. [42].

#### 2.2.6. Monitoring of Adverse Events

Adverse events encompassed any undesirable experiences encountered by a participant during the study, irrespective of their relation to trial procedures or the stepped‐care program. All adverse events, whether self‐reported during an assessment or detected by helpers during a session, were documented. Protocols were developed to address the detection of risk situations, including suicide risk, gender‐based violence, and extreme anxiety.

#### 2.2.7. Sample Size

Based on prior studies on PM+ [43, 44], we aimed to detect a Cohen’s *d* effect size of 0.3 (small‐to‐moderate effect size) in the intervention group for our primary outcome. Power calculations for a repeated measures design indicated a required sample size of *N* = 74 per group (power = 0.95, alpha = 0.05, two‐sided, and rho = 0.9). Considering an estimated 30% attrition rate, the initial target sample size was set at *N* = 212 (106 participants per group).

#### 2.2.8. Data Analysis

The stepped‐care program was analyzed as a unified intervention without distinguishing between participants who received PM+ and those who completed their intervention after DWM. Missing data were not imputed. The main analysis followed an intention‐to‐treat (ITT) approach, with robust standard errors to estimate the treatment effect on mental health outcomes. Additionally, sensitivity analyses were conducted to evaluate the robustness of the findings.

##### 2.2.8.1. ITT Analysis

A pre‐specified ITT analysis was conducted for both primary and secondary outcomes, including all randomized participants. The primary outcome analysis assessed the between‐group difference in depressive and anxiety symptoms (measured using the PHQ‐ADS) at T2, T3, and T4, with T4 (week 21) being the primary measure of interest. To estimate the treatment effect at each time point, linear mixed‐effects models were used. The models included group (intervention vs. control), time (T2–T4), and their interaction as fixed effects and a random intercept for participants to account for within‐person correlation due to repeated measures. Baseline outcome scores (PHQ‐ADS) were added as covariates to adjust for initial symptom severity and improve the precision of treatment effect estimates. From these models, estimated marginal means (EMMs) at each time point with their 95% confidence intervals (CIs) were obtained, and between‐group differences in EMMs were computed at T2, T3, and T4. Standardized effect sizes (SESs) were calculated as the pairwise differences between EMMs divided by the model’s residual standard deviation (SD), enabling interpretation of effect magnitude on a common scale. Further ITT analyses were conducted to estimate the treatment effect on secondary outcomes, including depressive symptoms (PHQ‐9), anxiety symptoms (GAD‐7), PTSD symptoms (PCL‐5), and HR‐QoL (EQ‐5D‐5L), using the same model specification as described above. To assess the robustness of the findings against potential model misspecifications, fully adjusted models were run, incorporating age, gender, educational level, and job facility as covariates.

##### 2.2.8.2. Sensitivity Analysis

Per‐protocol and complete‐case analyses were conducted for primary and secondary outcomes. The per‐protocol analysis was pre‐specified and included all participants in the control group and those in the intervention group that met the adherence criteria. Adherence criteria were defined as those who (a) attended at least 3 out of 5 DWM support sessions (by phone or message) and (b) if applicable (based on the K10 score at T2), attended at least 4 out of 5 PM+ sessions. The complete‐case analysis included only participants who completed all study assessments.

##### 2.2.8.3. Software

All statistical analyses were conducted using R 4.4.2 (R Core [45]). The tableone package [46] was used to compute the descriptives. Linear mixed models were computed with the nlme package [47, 48], while robust standard errors and EMMs were obtained using the clubSandwich [49] and emmeans [50] packages, respectively.

## 3. Results

A total of 165 participants were included in the study (intervention group: *N* = 83; control group: *N* = 82), which accounts for 78% of the initially targeted sample size. Of the 165 randomized participants, 128 completed the follow‐up (T4), resulting in an attrition rate of 22.4%. The complete participant flow diagram can be seen in Figure 2. Within the intervention group, of the 62 participants who completed T2 after DWM, 57 (92%) stepped up to PM+. K10 data is reported in the Supporting Information.

**Figure 2:**
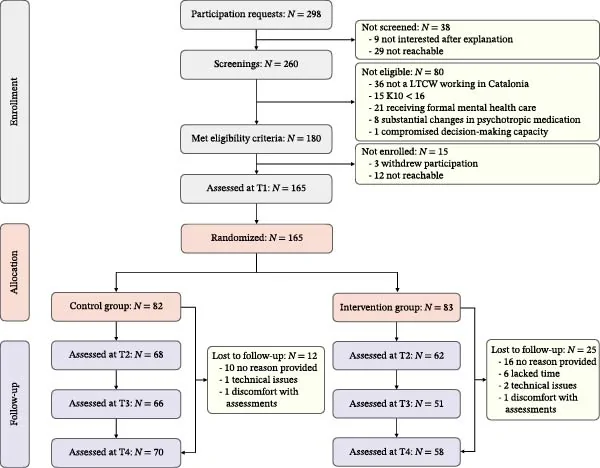
CONSORT‐SPI participants flow diagram. K10, Kessler Psychological Distress Scale.

The participants were predominantly women (95.8%), with a mean age of 42.2 years. Most worked in long‐term care facilities (80%). The most common job role was geriatric care assistance (28.5%), followed by nursing assistance (13.9%), administrative management (10,3%), nursing (7.9%), caregiving without formal training (7.3%), and social work (6.1%). The full details on the participants’ characteristics at baseline are shown in Table 1.

**Table 1:** Participants’ characteristics at baseline (T1).

Variable	Overall *N* = 165	Group		*p*‐Value
		Control *n* = 82	Intervention *n* = 83	
Age, *M* (SD)	42.2 (10.8)	41.9 (11.0)	42.5 (10.8)	0.722
Gender, *n* (%)	—	—	—	0.987
Female	158 (95.8%)	78 (95.1%)	80 (96.4%)	—
Male	7 (4.2%)	4 (4.9%)	3 (3.6%)	—
Educational level, *n* (%)	—	—	—	0.032
Primary	6 (3.6%)	1 (1.2%)	5 (6.0%)	—
Secondary	16 (9.7%)	9 (11.0%)	7 (8.4%)	—
Technical/professional	68 (41.2%)	27 (32.9%)	41 (49.4%)	—
University	75 (45.5%)	45 (54.9%)	30 (36.1%)	—
Job type, *n* (%)	—	—	—	0.029
Nursing	13 (7.9%)	6 (7.3%)	7 (8.4%)	—
Nursing assistance	23 (13.9%)	8 (9.8%)	15 (18.1%)	—
Geriatric care assistance	47 (28.5%)	23 (28.0%)	24 (28.9%)	—
Caregiving without formal training	13 (7.9%)	6 (7.3%)	7 (8.4%)	—
Physiotherapy	5 (3.0%)	4 (4.9%)	1 (1.2%)	—
Psychology	8 (4.8%)	6 (7.3%)	2 (2.4%)	—
Occupational therapy	5 (3.0%)	3 (3.7%)	2 (2.4%)	—
Social work	10 (6.1%)	2 (2.4%)	8 (9.6%)	—
Administrative management	17 (10.3%)	14 (17.1%)	3 (3.6%)	—
Others	24 (14.5%)	10 (12.2%)	14 (16.9%)	—
Job facility, *n* (%)	—	—	—	0.842
Residency	118 (71.5%)	60 (73.2%)	58 (69.9%)	—
Sociosanitary centre	14 (8.5%)	6 (7.3%)	8 (9.6%)	—
Home‐care	33 (20.0%)	16 (19.5%)	17 (20.5%)	—
Outcomes scores, *M* (SD)				
PHQ‐ADS	19.7 (9.9)	18.3 (9.8)	21 (9.9)	0.084
PHQ‐9	9.7 (5.7)	8.9 (5.7)	10.5 (5.7)	0.075
GAD‐7	10 (4.7)	9.4 (4.6)	10.5 (4.8)	0.142
PCL‐5	12.3 (7.2)	11.4 (7.0)	13.2 (7.3)	0.103
EQ‐5D‐5L	0.8 (0.1)	0.8 (0.1)	0.8 (0.1)	0.139

Abbreviations: EQ‐5D‐5L, EuroQol 5‐dimensional descriptive system–5‐level version (measures health‐related quality of life); GAD‐7, 7‐item Generalized Anxiety Disorder scale; *M*, mean; PCL‐5, PTSD checklist for DSM‐5; PHQ‐9, 9‐item Patient Health Questionnaire (measures depressive symptoms); PHQ‐ADS, Patient Health Questionnaire‐Anxiety and Depression Scale; SD, standard deviation.

Results from fully adjusted models (accounting for age, gender, educational level, and job facility) are reported as follows. Both baseline‐adjusted and fully adjusted models are presented in Table 2. The primary outcome (i.e., the between‐group difference in depressive and anxiety symptoms at T4, measured using the PHQ‐ADS) was statistically significant, favoring the intervention group, with a mean difference of 4.2 (95% CI: 1.1–7.3) and a moderate to large effect size (SES = 0.8, 95% CI: 0.1–1.4). Significant differences were also observed at T2 (mean difference = 3.4, 95% CI: 1.1–5.7; SES = 0.6, 95% CI: 0.1–1.1) and at T3 (mean difference = 5.8, 95% CI: 3.0–8.6; and SES = 1.0, 95% CI: 0.4–1.7). Secondary outcomes also showed significant differences when considering depressive and anxiety symptoms separately at all timepoints. For depressive symptoms, mean differences ranged from 2.0 (95% CI: 0.8–3.3) at T2 to 2.8 (95% CI: 1.2–4.4) at T3, and for anxiety symptoms, from 1.3 (95% CI: 0.1–2.5) at T2 to 2.9 (95% CI: 1.6–4.3) at T3. At T3 and T4, effect sizes ranged from moderate to large for both depressive and anxiety symptoms, with the largest effects observed for anxiety at T3 (SES = 1.0, 95% CI: 0.3–1.8). Regarding PTSD symptoms, no significant differences were observed at T2. However, at T3 and T4, the intervention group showed significantly lower symptom levels than the control group (T3: mean difference = 2.9, 95% CI: 0.8–4.9; T4: mean difference = 2.4, 95% CI: 0.3–4.6), with moderate effect sizes. No differences were found between groups in HR‐QoL at any timepoint. Figure 3 illustrates the trends over time in EMMs for primary and secondary outcomes with their CIs. Sensitivity analyses, including per‐protocol and complete‐case approaches, yielded results consistent with the main ITT analysis, supporting the robustness of the findings. Full details of the sensitivity analyses are provided in the Supporting Information.

**Figure 3:**
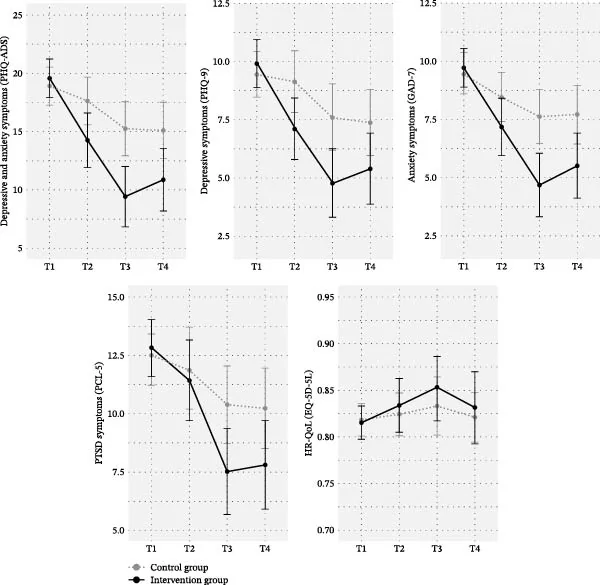
Fully‐adjusted estimated marginal means and 95% confidence intervals for primary and secondary outcomes over time (T1–T4). GAD‐7, 7‐item Generalized Anxiety Disorder scale; HR‐QoL, health‐related quality of life; PHQ‐9, 9‐item Patient Health Questionnaire; PHQ‐ADS, Patient Health Questionnaire–Anxiety and Depression Scale; PTSD, post‐traumatic stress disorder; PCL‐5, PTSD checklist for DSM‐5; EQ‐5D‐5L, EuroQol 5‐dimensional descriptive system–5‐level version.

**Table 2:** Estimated marginal means and standardized effect sizes (SES) of primary and secondary outcomes.

Outcome	Baseline‐adjusted models				Fully adjusted models^a^			
	Control	Intervention	Difference	SES	Control	Intervention	Difference	SES
Depressive and anxiety symptoms^b^								
T2	18.3 (17.0, 19.6)	14.8 (12.9, 16.6)	3.5 **(1.2, 5.7)**	0.6 (0.1, 1.1)	17.6 (15.5, 19.6)	14.2 (11.9, 16.6)	3.4 **(1.1, 5.7)**	0.6 (0.1, 1.1)
T3	15.9 (14.2, 17.5)	9.9 (7.8, 12.1)	5.9 **(3.2, 8.6)**	1.1 (0.3, 1.8)	15.2 (12.9, 17.5)	9.4 (6.8, 12.0)	5.8 **(3.0, 8.6)**	1.0 (0.4, 1.7)
T4 (primary outcome)	15.7 (13.7, 17.7)	11.4 (9.2, 13.6)	4.3 **(1.3, 7.4)**	0.8 (0.1, 1.5)	15.1 (12.7, 17.5)	10.8 (8.2, 13.5)	4.2 **(1.1, 7.3)**	0.8 (0.1, 1.4)
Depressive symptoms^b^								
T2	9.3 (8.5, 10.1)	7.2 (6.3, 8.2)	2.1 **(0.9, 3.3)**	0.7 (0.2, 1.1)	9.1 (7.8, 10.5)	7.1 (5.8, 8.4)	2.0 **(0.8, 3.3)**	0.6 (0.2, 1.1)
T3	7.8 (6.7, 8.8)	4.9 (3.7, 6.0)	2.9 **(1.3, 4.4)**	0.9 (0.3, 1.5)	7.6 (6.2, 9.0)	4.8 (3.3, 6.2)	2.8 **(1.2, 4.4)**	0.9 (0.3, 1.5)
T4	7.5 (6.4, 8.6)	5.5 (4.3, 6.7)	2.0 **(0.4, 3.7)**	0.6 (0.1, 1.2)	7.4 (6.0, 8.8)	5.4 (3.9, 6.9)	2.0 **(0.2, 3.7)**	0.6 (0.0, 1.2)
Anxiety symptoms^b^								
T2	8.9 (8.2, 9.6)	7.6 (6.6, 8.6)	1.3 **(0.1, 2.5)**	0.5 (−0.1, 1.0)	8.5 (7.4, 9.5)	7.2 (6.0, 8.4)	1.3 **(0.1, 2.5)**	0.4 (−0.0, 0.9)
T3	8.1 (7.3, 8.9)	5.1 (4.0, 6.2)	3.0 **(1.6, 4.4)**	1.1 (0.2, 2.0)	7.6 (6.5, 8.8)	4.7 (3.3, 6.1)	2.9 **(1.6, 4.3)**	1.0 (0.3, 1.8)
T4	8.2 (7.2, 9.2)	5.9 (4.8, 7.1)	2.2 **(0.7, 3.8)**	0.8 (0.0, 1.6)	7.7 (6.5, 9.0)	5.5 (4.1, 6.9)	2.2 **(0.7, 3.7)**	0.8 (0.1, 1.5)
PTSD symptoms^b^								
T2	11.8 (10.4, 13.1)	11.1 (9.6, 12.5)	0.7 (−1.3, 2.6)	0.2 (−0.3, 0.6)	12.0 (10.2, 13.7)	11.4 (9.7, 13.2)	0.5 (−1.5, 2.5)	0.1 (−0.4, 0.6)
T3	10.2 (8.9, 11.5)	7.1 (5.6, 8.7)	3.0 **(1.0, 5.1)**	0.7 (0.0, 1.4)	10.4 (8.7, 12.1)	7.5 (5.7, 9.4)	2.9 **(0.8, 4.9)**	0.7 (0.1, 1.3)
T4	10.0 (8.6, 11.4)	7.4 (5.8, 9.0)	2.6 **(0.4, 4.7)**	0.6 (−0.0, 1.3)	10.2 (8.5, 12.0)	7.8 (5.9, 9.7)	2.4 **(0.3, 4.6)**	0.6 (−0.0, 1.2)
HR‐QoL^b^								
T2	0.8 (0.8, 0.8)	0.8 (0.8, 0.9)	−0.0 (−0.0, 0.0)	−0.1 (−0.7, 0.4)	0.8 (0.8, 0.8)	0.8 (0.8, 0.9)	−0.0 (−0.0, 0.0)	−0.1 (−0.6, 0.3)
T3	0.8 (0.8, 0.9)	0.8 (0.8, 0.9)	−0.0 (−0.1, 0.0)	−0.2 (−1.1, 0.6)	0.8 (0.8, 0.9)	0.9 (0.8, 0.9)	−0.0 (−0.1, 0.0)	−0.2 (−0.9, 0.4)
T4	0.8 (0.8, 0.8)	0.8 (0.8, 0.9)	−0.0 (−0.1, 0.0)	−0.1 (−0.8, 0.6)	0.8 (0.8, 0.8)	0.8 (0.8, 0.9)	−0.0 (−0.1, 0.0)	−0.1 (−0.7, 0.5)

*Note:* Bold prints indicate statistically significant differences.

Abbreviations: HR‐QoL, health‐related quality of life; PTSD, post‐traumatic stress disorder; SES, standardized effect sizes.

^a^Adjusted for age, gender, educational level, and job facility.

^b^The number of participants at T2, T3, and T4 was 68, 66, and 70 in the control group, and 62, 51, and 58 in the intervention group.

Treatment fidelity assessments indicated strong adherence to the protocol, with fidelity rates ranging from 80% to 100% for DWM and 70% to 100% for PM+. The suicide risk protocol was activated for 10 participants with suicide ideation (4 in the control group and 6 in the intervention group), none of whom were assessed as a high or imminent risk. In all cases, participants were referred to mental health services. No other serious adverse events were reported, and adverse events were not considered related to study participation.

## 4. Discussion

We evaluated the effectiveness of a digital stepped‐care program, adapted from two WHO psychological interventions (DWM and PM+), in reducing anxiety and depressive symptoms among LTCWs experiencing psychological distress. Our findings demonstrate that the program consistently led to significant reductions in anxiety and depressive symptoms (examined together as the primary outcome and separately) across all study time points, with moderate to large effect sizes. Furthermore, the program also led to significant reductions in PTSD symptoms at the post‐program time points, with moderate effect sizes. However, no effects were observed on HR‐QoL at any time point. Sensitivity analyses confirmed the robustness of these findings, further supporting the validity of the program’s effects. In terms of acceptability, no serious adverse events related to the program were detected, and participant retention was relatively high throughout the study, reinforcing its applicability.

To the best of our knowledge, this is the first study to consistently demonstrate positive effects in reducing depressive, anxiety, and PTSD symptoms through a digital intervention for LTCWs. A previous study evaluated the effectiveness of DWM as a standalone intervention for this population during the COVID‐19 pandemic [17]. It compared DWM to an alternative activity (reading a short novel) and assessed its impact on anxiety and PTSD symptoms, but the findings were mixed. An initial analysis [17], using continuous outcome measures as in our study, found no significant effects. A subsequent secondary analysis of the same dataset [51], which used dichotomized outcomes, reported statistically significant effects, although these emerged primarily in per‐protocol analyses, with more limited evidence in the ITT analyses. Several methodological differences may account for the more robust and consistent results observed in our study. The intervention in the Riello et al. [17] study was only minimally adapted, did not incorporate structured emotional support, and did not include a control group. In contrast, our study involved a rigorous adaptation process to tailor the program to the specific needs and characteristics of the target population, a factor identified as critical for intervention success [52, 53]. Additionally, we included structured emotional support through the emotional management facilitator or helper, which likely contributed to the intervention’s effectiveness, as professional guidance has been associated with greater effect sizes in digital mental health interventions [54]. These distinctions suggest that the success of digital mental health interventions depends not only on their content, but also on the way they are adapted to the target population and implemented.

Another key difference is that we implemented a stepped‐care approach, offering PM+ as a second, higher‐intensity intervention to participants who continued to experience psychological distress after completing DWM. Stepped‐care formats have been associated with greater scalability, as they allow individuals to begin with a low‐intensity intervention and only escalate to a higher level of care if necessary, thereby expanding access to treatment while utilizing fewer resources [55]. However, the fact that 92% of our participants stepped up to PM+ raises the possibility that the robust positive effects observed in our study may be primarily attributable to PM+. This is a relevant point to explore as a program based solely on PM+ would require less intervention time and lower costs. In the systematic review and meta‐analysis by Schäfer et al. [56], 23 randomized controlled trials evaluating PM+ as a standalone intervention were analyzed, covering diverse contexts and populations facing adversity. While the review found positive effects of the intervention on depression, anxiety, and PTSD symptoms, the effect sizes were smaller than those observed in our study, ranging from small to moderate. One possible explanation for our larger effect sizes is the use of a stepped‐care approach, which may have had a cumulative and enhancing effect on outcomes. Although direct comparisons cannot be made, the sequential delivery of DWM followed by PM+ may have progressively amplified the benefits, reinforcing and sustaining symptom reduction over time. Further research is needed to better understand the potential additive effects of this stepped‐care approach.

DWM and PM+ are both supported by extensive evidence as standalone interventions, with their ACT‐ and CBT‐based content, respectively, showing effectiveness in improving mental health outcomes in populations facing adversity [56–59]. However, few studies have evaluated these interventions together within a stepped‐care approach, as we did. Notably, two previous studies that also evaluated the effectiveness of DWM and PM+ on depression, anxiety, and PTSD symptoms within a stepped‐care program yielded similar results [22, 23]. While these studies shared a comparable methodological design with ours, they were conducted in different populations, one with healthcare workers and the other with migrants, and during an earlier phase of the COVID‐19 pandemic (between November 2021 and April 2023). These consistent findings across studies highlight the program’s effectiveness in diverse settings and populations and underscore its potential for broader implementation.

Finally, it is important to consider why a reduction in anxiety, depressive, and PTSD symptoms was not followed by an improvement in HR‐QoL. Several theories have been proposed to explain why symptom reduction or remission does not always translate into functional recovery or improvements in positive mental health outcomes. One explanation is that changes in these broader outcomes may take longer to emerge as improvements often lag behind symptom reduction [60]. Supporting this view, a meta‐analysis of 59 randomized controlled trials of CBT for anxiety disorders observed that longer treatments may be required to produce meaningful gains in quality of life [61]. Another perspective holds that many individuals continue to live under adverse circumstances during and after treatment, which may hinder improvements in daily functioning and subjective well‐being. This is consistent with the broader discussion around what should be included in the concept of HR‐QoL, which, despite variations in definitions, is widely recognized as an inherently multidimensional construct [62]. Keyes [63] dual continuum model provides a theoretical framework for this perspective, emphasizing that mental illness and positive mental health should not be viewed as merely opposite ends of the same spectrum. Instead, they represent interrelated but distinct phenomena. Consequently, our findings suggest that individual‐level mental health interventions alone may not be sufficient. Broader system‐level efforts, such as workplace interventions and public policies aimed at improving working conditions, remain essential to sustainably promote the mental health and well‐being of LTCWs across all relevant outcomes. As highlighted by recent recommendations, the impact of psychosocial work environments on mental health must be addressed through structural changes, particularly for those facing precarious working conditions [2].

Our study has certain limitations. First, we did not reach our planned sample size. Recruitment was stopped in accordance with the predefined study timeline as an extension was not feasible. Nevertheless, our final sample was sufficient to detect statistically significant effects with robust effect sizes. The relatively high participant retention may have contributed to maintaining adequate statistical power. Second, a blinded design was not feasible due to the nature of the psychological interventions and protocol requirements. Third, all outcomes were based on self‐reported measures. This approach can introduce variability and may be subject to response biases. Fourth, adverse events were systematically monitored throughout the study but were not incorporated into the analysis. Although they were infrequent (*n* = 10), they might have influenced the study outcomes. Fifth, our sample was predominantly composed of women. While this reflects the gender distribution within the LTCW population, it should be taken into account when considering the generalizability of the findings. Sixth, we observed higher attrition in the intervention group compared to the control group, which may have affected the representativeness of follow‐up data. However, sensitivity analyses confirmed the robustness of our findings. Lastly, the study had a relatively short follow‐up period of 21 weeks. As such, we cannot determine whether the positive effects observed would be sustained or diminish over a longer period of time.

## 5. Conclusions

This study provides evidence supporting the effectiveness of a digital and scalable stepped‐care program to support LTCWs’ mental health. Our robust results can already inform occupational and public health actions and guide decision‐making among policymakers. This does not, however, replace the need for broader interventions that go beyond the individual level and address structural and workplace‐related factors.

**Nomenclature**
ACT: Acceptance and commitment therapy
CBT: Cognitive–behavioral therapy
CI: Confidence interval
DSM‐5: Diagnostic and Statistical Manual of Mental Disorders, 5^th^ Edition
DWM: Doing What Matters in Times of Stress
EMM: Estimated marginal means
EQ‐5D‐5L: EuroQol 5‐dimensional descriptive system–5‐level version
GAD‐7: Generalized anxiety disorder scale
HR‐QoL: Health‐related quality of life
ITT: Intention‐to‐treat
K10: Kessler Psychological Distress Scale
LTCWs: Long‐term care workers
PCL‐5: PTSD checklist for DSM‐5
PFA: Psychological First Aid
PHQ‐9: Patient Health Questionnaire
PHQ‐ADS: Patient Health Questionnaire–Anxiety and Depression Scale
PM+: Problem Management Plus
PTSD: Post‐traumatic stress disorder
REDCap: Research electronic data capture
SD: Standard deviation
SES: Standardized effect size
SH+: Self‐Help Plus
WHO: World Health Organization.

## Author Contributions

**Leticia González-Spinoglio:** data curation, formal analysis, investigation, methodology, project administration, software, visualization, writing – original draft, writing – review and editing. **Mireia Esplugues-Tormo**: data curation, investigation, writing – review and editing. **Iago Giné-Vázquez**: formal analysis, software, validation, writing – review and editing. **Jordi Rodeiro-Boliart**: formal analysis, software, writing – review and editing. **Salvatore Aguilar-Ortiz**: investigation, writing – review and editing. **Jose Luis Ayuso-Mateos and Marit Sijbrandij**: conceptualization, funding acquisition, methodology, writing – review and editing. **Kerry R. McGreevy, Roberto Mediavilla, and Anke B. Witteveen**: methodology, writing – review and editing. **Rinske Roos**: methodology, software, writing – review and editing. **Josep Maria Haro**: conceptualization, funding acquisition, methodology, project administration, resources, supervision, writing – review and editing. **Mireia Felez-Nobrega**: conceptualization, data curation, investigation, methodology, project administration, supervision, validation, writing – review and editing.

## Funding

This work was supported by the Fundació La Marató de TV3 (Grant 1114/C/2021). Leticia González‐Spinoglio was supported by the Instituto de Salud Carlos III, in the form of a pre‐doctoral grant (PFIS‐Grant, Code FI23/00015). Mireia Esplugues‐Tormo was supported by the Investigo AGAUR Program contract, funded by the European Union NextGenerationEU (Code 100031TG3).

## Disclosure

After using the ChatGPT, the authors reviewed and edited the content as needed and take full responsibility for the content of the publication. The funders were not involved in the study design, data collection and management, decision to publish, or the preparation of the manuscript.

## Ethics Statement

Research was approved by the Ethics Committee of Parc Sanitari Sant Joan de Deu (ID: PIC‐214‐21).

## Conflicts of Interest

The authors declare no conflicts of interest.

## Supporting Information

Additional supporting information can be found online in the Supporting Information section.

## Supporting information


**Supporting Information** All supporting information is included in a single file titled “5875889.f1.docx”. This file contains a detailed description of the REPICAL stepped‐care program, K10 scores, and the results of per‐protocol and complete‐case analyses. Figure S1: Images of the DWM REPICAL app. Table S1: Kessler Psychological Distress Scale (K10) scores. Figure S2: EMM and 95% CIs for primary and secondary outcomes over time (T1–T4) from the per‐protocol analysis. Table S2: EMM and SES of primary and secondary outcomes from the per‐protocol analysis. Figure S3: EMM and 95% CIs for primary and secondary outcomes over time (T1–T4) from the complete‐case analysis. Table S3: EMM and SES of primary and secondary outcomes from the complete‐case analysis.

## Data Availability

The data that support the findings of this study are available upon request from the corresponding author. The data are not publicly available due to privacy or ethical restrictions.
