# Supplementary material for: A Randomized Controlled Trial of a Digital Stepped‐Care Program to Reduce Anxiety and Depressive Symptoms Among Long‐Term Care Workers With Psychological Distress: The REPICAL Study
Source: Depress Anxiety. 2026 Oct 2;2026:5875889. doi: 10.1155/da/5875889 (PMC13633456; doi:10.1155/da/5875889)
Supplement: Supplementary file 1 [file DA-2026-5875889-s001.docx]

A Randomized Controlled Trial of a Digital Stepped-Care Program to Reduce Anxiety and Depressive Symptoms Among Long-Term Care Workers With Psychological Distress: The REPICAL study

**Supporting information**

**Contents**

REPICAL Stepped-care program, p. 3

Kessler Psychological Distress Scale scores, p. 5

Per-protocol analyses, p. 5

Complete-case analyses, p. 8

**Abbreviations**

ACT: Acceptance and commitment therapy

CBT: cognitive-behavioral therapy

DWM: Doing What Matters in Times of Stress

EQ-5D-5L: EuroQol 5-Dimensional descriptive system – 5-Level version

GAD-7: Generalized Anxiety Disorder scale

HR-QoL: Health-related quality of life

K10: Kessler Psychological Distress Scale

PHQ-9: Patient Health Questionnaire

PHQ-ADS: Patient Health Questionnaire-Anxiety and Depression Scale

PM+: Problem Management Plus

PTSD: Post-traumatic Stress Disorder

SES: Standardized effect sizes

SH+: Self-Help Plus

WHO: World Health Organization

**REPICAL stepped-care program**

**Step 1: Doing what matters in times of stress (DWM)**

This intervention is an adaptation of *Doing What Matters in Times of Stress: An Illustrated Guide* (World Health Organization [WHO], 2020), which is part of the WHO stress management program *Self-Help Plus* (SH+) (WHO, 2021). DWM is based on acceptance and commitment therapy (ACT). The intervention consists of five modules, accessed through the app/web, each addressing techniques and concepts aimed at enhancing stress management. The five modules are: “Grounding”, “Unhooking”, “Acting on your values”, “Being kind” and “Making room”. The intervention spans a total of 5 weeks, with each module designed for completion within one week. New modules unlock weekly, providing reading content, images, and audio recordings on new ACT techniques. To encourage participation and motivation, modules include questions, and at the end of each module, participants are invited to complete a practice planning. Although this is a self-guided intervention, each participant was assigned an emotional management facilitator ("helper”) who provided support during the intervention. This support included a 15-minute weekly phone call, one for each module, scheduled in advance with the participant. During these calls, participants could address any doubts or concerns related to module content and exercise practices. Additionally, participants had the option to communicate asynchronously with their helper through a platform chat in case they needed to reschedule calls or report technical incidents. This communication option was also particularly beneficial for holding the weekly support sessions with participants who faced scheduling challenges. Figure S1 shows images of the DWM REPICAL app.

**Step 2: Problem Management Plus (PM+).**

This step was exclusively for participants in the intervention group who, after completing DWM, still met the criteria for psychological distress (K10 ≥ 16). This intervention is an adapted version of the WHO's *Problem Management Plus (PM+): Individual Psychological Help for Adults Impaired by Distress in Communities Exposed to Adversity* (WHO, 2016), grounded in Cognitive-Behavioral Therapy (CBT). It comprises five 60-minute individual videoconference sessions, each focusing on different CBT techniques, with the primary goal of acquiring effective tools for problem management. These sessions are: “Managing stress”, “Managing problem”, “Get going, keep doing”, “Strengthening social support” and “Staying well”. These sessions, conducted by the participant's helper, took place weekly, totaling a 5-week duration.

**Figure S1.** Images of the DWM REPICAL app.

**Kessler Psychological Distress Scale scores**

Descriptive Kessler Psychological Distress Scale (K10) scores at screening and T2 are presented in Table S1.

| **Table S1.**  Kessler Psychological Distress Scale (K10) scores | | | |
| --- | --- | --- | --- |
|  | **Overall** | **Control** | **Intervention** |
| **K10 score, M (SD)** |  |  |  |
| T0 (Screening) | 27.8 (6.7) | 27,2 (7,0) | 28,4 (6,4) |
| T2 | 25.8 (7.8) | 25,5 (8,2) | 26,1 (7,4) |
| K10, Kessler Psychological Distress Scale; M, mean; SD, standard deviation. **Note.** Only participants who met all eligibility criteria during screening were included in this analysis. The number of participants at T0 and T2 was 165 and 130 overall, 82 and 68 in the control group, and 83 and 62 in the intervention group. | | | |

**Per-protocol analysis**

Per-protocol analysis included all participants in the control group and those in the intervention group that met the adherence criteria. Adherence criteria was defined as those who: (a) attended at least 3 out of 5 DWM support sessions (by phone or message), and (b) if applicable (based on the K10 score at T2), attended at least 4 out of 5 PM+ sessions. The results from the per-protocol analysis are presented in Figure S2 and Table S2.

**Figure S2. Per-protocol analysis.** Fully-adjusted estimated marginal means and 95% confidence intervals for primary and secondary outcomes over time (T1–T4). PHQ-ADS, Patient Health Questionnaire-Anxiety and Depression Scale; PHQ-9, 9-item Patient Health Questionnaire; GAD-7, 7-item Generalized Anxiety Disorder scale; PTSD, post-traumatic stress disorder; PCL-5, PTSD Checklist for DSM-5; HR-QoL, health-related quality of life. EQ-5D-5L, EuroQol 5-dimensional descriptive system – 5-level version.

| **Table S2. Per-protocol analysis**  Estimated marginal means and standardized effect sizes (SES) of primary and secondary outcomes | | | | | | | | |  |
| --- | --- | --- | --- | --- | --- | --- | --- | --- | --- |
|  | **Baseline-adjusted models** | | | | **Fully adjusted models*** | | | | |
|  | **Control** | **Intervention** | **Difference** | **SES** | **Control** | **Intervention** | **Difference** | **SES** | |
| **Depressive and anxiety symptoms†** |  |  |  |  |  |  |  |  | |
| T2 | 18.2 (16.9, 19.5) | 14.1 (12.0, 16.3) | 4.1 (1.6, 6.6) | 0.7 (0.1, 1.4) | 17.8 (15.6, 20.0) | 13.8 (11.0, 16.6) | 4.0 (1.4, 6.5) | 0.7 (0.1, 1.3) | |
| T3 | 15.8 (14.2, 17.5) | 9.7 (7.4, 11.9) | 6.2 (3.3, 9.0) | 1.1 (0.3, 2.0) | 15.4 (12.9, 17.8) | 9.3 (6.4, 12.2) | 6.0 (3.1, 9.0) | 1.1 (0.4, 1.8) | |
| T4 (primary outcome) | 15.7 (13.7, 17.7) | 11.2 (8.8, 13.7) | 4.5 (1.3, 7.7) | 0.8 (0.0, 1.6) | 15.2 (12.7, 17.7) | 10.9 (7.8, 13.9) | 4.4 (1.0, 7.7) | 0.8 (0.1, 1.5) | |
| **Depressive symptoms†** |  |  |  |  |  |  |  |  | |
| T2 | 9.3 (8.5, 10.0) | 6.9 (5.8, 8.0) | 2.4 (1.0, 3.7) | 0.7 (0.2, 1.3) | 9.2 (7.8, 10.7) | 6.9 (5.4, 8.5) | 2.3 (0.9, 3.7) | 0.7 (0.2, 1.2) | |
| T3 | 7.7 (6.7, 8.7) | 4.7 (3.5, 5.9) | 3.0 (1.4, 4.6) | 0.9 (0.3, 1.6) | 7.7 (6.2, 9.2) | 4.8 (3.1, 6.4) | 2.9 (1.2, 4.7) | 0.9 (0.3, 1.5) | |
| T4 | 7.5 (6.4, 8.6) | 5.5 (4.1, 6.8) | 2.0 (0.2, 3.8) | 0.6 (0.0, 1.3) | 7.5 (6.0, 8.9) | 5.5 (3.8, 7.3) | 2.0 (0.0, 3.9) | 0.6 (-0.0, 1.2) | |
| **Anxiety symptoms†** |  |  |  |  |  |  |  |  | |
| T2 | 8.9 (8.2, 9.6) | 7.3 (6.2, 8.4) | 1.6 (0.3, 2.9) | 0.6 (-0.1, 1.3) | 8.5 (7.4, 9.7) | 7.0 (5.6, 8.4) | 1.5 (0.2, 2.9) | 0.6 (-0.0, 1.1) | |
| T3 | 8.1 (7.3, 8.9) | 5.0 (3.9, 6.1) | 3.1 (1.7, 4.5) | 1.1 (0.1, 2.1) | 7.7 (6.5, 8.9) | 4.7 (3.2, 6.2) | 3.0 (1.6, 4.5) | 1.1 (0.2, 1.9) | |
| T4 | 8.2 (7.2, 9.2) | 5.8 (4.6, 7.0) | 2.4 (0.8, 4.0) | 0.9 (-0.0, 1.8) | 7.8 (6.5, 9.1) | 5.5 (4.0, 7.0) | 2.3 (0.7, 3.9) | 0.8 (0.1, 1.6) | |
| **PTSD symptoms†** |  |  |  |  |  |  |  |  | |
| T2 | 11.7 (10.4, 13.1) | 10.6 (8.8, 12.4) | 1.2 (-1.1, 3.4) | 0.3 (-0.3, 0.8) | 12.1 (10.3, 13.9) | 11.2 (9.1, 13.3) | 0.9 (-1.4, 3.2) | 0.2 (-0.3, 0.8) | |
| T3 | 10.1 (8.8, 11.4) | 6.9 (5.3, 8.5) | 3.2 (1.2, 5.3) | 0.8 (0.0, 1.5) | 10.6 (8.8, 12.3) | 7.5 (5.6, 9.5) | 3.0 (0.9, 5.2) | 0.7 (0.1, 1.3) | |
| T4 | 10.0 (8.6, 11.4) | 7.2 (5.5, 9.0) | 2.7 (0.5, 5.0) | 0.6 (-0.1, 1.3) | 10.4 (8.6, 12.2) | 7.9 (5.8, 9.9) | 2.5 (0.3, 4.8) | 0.6 (-0.0, 1.2) | |
| **HR-QoL†** |  |  |  |  |  |  |  |  | |
| T2 | 0.8 (0.8, 0.8) | 0.8 (0.8, 0.9) | -0.0 (-0.1, 0.0) | -0.2 (-0.9, 0.4) | 0.8 (0.8, 0.8) | 0.8 (0.8, 0.9) | -0.0 (-0.1, 0.0) | -0.2 (-0.7, 0.3) | |
| T3 | 0.8 (0.8, 0.9) | 0.8 (0.8, 0.9) | -0.0 (-0.1, 0.0) | -0.3 (-1.1, 0.5) | 0.8 (0.8, 0.9) | 0.9 (0.8, 0.9) | -0.0 (-0.1, 0.0) | -0.3 (-0.9, 0.3) | |
| T4 | 0.8 (0.8, 0.8) | 0.8 (0.8, 0.9) | -0.0 (-0.1, 0.1) | -0.0 (-0.7, 0.6) | 0.8 (0.8, 0.8) | 0.8 (0.8, 0.9) | -0.0 (-0.1, 0.0) | -0.0 (-0.7, 0.6) | |
| * Adjusted for age, gender, educational level, and job facility.  **†** The number of participants at T2, T3 and T4 was 68, 66 and 70 in the control group, and 45, 45 and 45 in the intervention group.  SES, standardized effect sizes; PTSD, post-traumatic stress disorder; HR-QoL, health-related quality of life. | | | | | | | | |  |

**Complete-case analysis**

Complete-case analysis included only participants who completed all study assessments. The results from the complete-case analysis are presented in Figure S3 and Table S3.

**Figure S3. Complete-case analysis.** Fully-adjusted estimated marginal means and 95% confidence intervals for primary and secondary outcomes over time (T1–T4). PHQ-ADS, Patient Health Questionnaire-Anxiety and Depression Scale; PHQ-9, 9-item Patient Health Questionnaire; GAD-7, 7-item Generalized Anxiety Disorder scale; PTSD, post-traumatic stress disorder; PCL-5, PTSD Checklist for DSM-5; HR-QoL, health-related quality of life. EQ-5D-5L, EuroQol 5-dimensional descriptive system – 5-level version.

| **Table S3. Complete-case analysis**  Estimated marginal means and standardized effect sizes (SES) of primary and secondary outcomes | | | | | | | | |  |
| --- | --- | --- | --- | --- | --- | --- | --- | --- | --- |
|  | **Baseline-adjusted models** | | | | **Fully adjusted models*** | | | | |
|  | **Control** | **Intervention** | **Difference** | **SES** | **Control** | **Intervention** | **Difference** | **SES** | |
| **Depressive and anxiety symptoms†** |  |  |  |  |  |  |  |  | |
| T2 | 18.1 (16.8, 19.5) | 14.0 (12.1, 16.0) | 4.1 (1.7, 6.5) | 0.7 (0.2, 1.3) | 17.8 (15.5, 20.1) | 14.0 (11.2, 16.7) | 3.9 (1.4, 6.3) | 0.7 (0.2, 1.2) | |
| T3 | 15.6 (14.0, 17.2) | 9.6 (7.5, 11.7) | 6.0 (3.3, 8.7) | 1.1 (0.4, 1.8) | 15.3 (12.8, 17.8) | 9.5 (6.7, 12.4) | 5.7 (2.9, 8.6) | 1.0 (0.4, 1.7) | |
| T4 (primary outcome) | 15.5 (13.4, 17.6) | 11.0 (8.6, 13.3) | 4.5 (1.4, 7.7) | 0.8 (0.1, 1.5) | 15.2 (12.6, 17.8) | 10.9 (7.9, 13.9) | 4.3 (1.0, 7.6) | 0.8 (0.1, 1.5) | |
| **Depressive symptoms†** |  |  |  |  |  |  |  |  | |
| T2 | 9.2 (8.4, 10.0) | 6.9 (5.9, 7.9) | 2.3 (1.0, 3.6) | 0.7 (0.2, 1.2) | 9.3 (7.8, 10.8) | 7.2 (5.6, 8.7) | 2.1 (0.8, 3.5) | 0.7 (0.2, 1.1) | |
| T3 | 7.6 (6.7, 8.6) | 4.7 (3.6, 5.9) | 2.9 (1.4, 4.4) | 0.9 (0.3, 1.5) | 7.7 (6.2, 9.3) | 5.0 (3.3, 6.6) | 2.7 (1.1, 4.4) | 0.9 (0.3, 1.4) | |
| T4 | 7.3 (6.2, 8.5) | 5.3 (4.0, 6.6) | 2.0 (0.3, 3.8) | 0.6 (0.0, 1.2) | 7.4 (5.9, 9.0) | 5.6 (3.8, 7.3) | 1.9 (0.0, 3.7) | 0.6 (-0.0, 1.2) | |
| **Anxiety symptoms†** |  |  |  |  |  |  |  |  | |
| T2 | 8.9 (8.1, 9.6) | 7.2 (6.1, 8.2) | 1.7 (0.4, 3.0) | 0.6 (-0.0, 1.3) | 8.6 (7.4, 9.7) | 6.9 (5.5, 8.3) | 1.6 (0.3, 2.9) | 0.6 (0.0, 1.1) | |
| T3 | 7.9 (7.1, 8.7) | 4.9 (3.9, 6.0) | 3.0 (1.7, 4.4) | 1.1 (0.2, 2.0) | 7.6 (6.3, 8.9) | 4.7 (3.2, 6.2) | 2.9 (1.5, 4.3) | 1.0 (0.3, 1.8) | |
| T4 | 8.1 (7.1, 9.2) | 5.7 (4.6, 6.8) | 2.4 (0.9, 4.0) | 0.9 (0.1, 1.7) | 7.8 (6.4, 9.2) | 5.5 (4.0, 7.0) | 2.3 (0.8, 3.9) | 0.8 (0.1, 1.5) | |
| **PTSD symptoms†** |  |  |  |  |  |  |  |  | |
| T2 | 11.7 (10.3, 13.1) | 10.4 (8.7, 12.1) | 1.3 (-0.9, 3.6) | 0.3 (-0.2, 0.9) | 12.2 (10.3, 14.1) | 11.1 (9.0, 13.1) | 1.1 (-1.2, 3.4) | 0.3 (-0.3, 0.8) | |
| T3 | 9.9 (8.5, 11.3) | 6.7 (5.1, 8.2) | 3.2 (1.1, 5.3) | 0.8 (0.1, 1.4) | 10.4 (8.6, 12.2) | 7.4 (5.5, 9.3) | 3.0 (0.8, 5.1) | 0.7 (0.1, 1.3) | |
| T4 | 10.3 (8.8, 11.8) | 6.9 (5.3, 8.6) | 3.3 (1.1, 5.5) | 0.8 (0.1, 1.5) | 10.7 (8.9, 12.6) | 7.7 (5.6, 9.7) | 3.1 (0.8, 5.3) | 0.7 (0.1, 1.3) | |
| **HR-QoL†** |  |  |  |  |  |  |  |  | |
| T2 | 0.8 (0.8, 0.8) | 0.8 (0.8, 0.9) | -0.0 (-0.0, 0.0) | -0.1 (-0.7, 0.4) | 0.8 (0.8, 0.9) | 0.8 (0.8, 0.9) | -0.0 (-0.0, 0.0) | -0.1 (-0.6, 0.4) | |
| T3 | 0.8 (0.8, 0.9) | 0.8 (0.8, 0.9) | -0.0 (-0.1, 0.0) | -0.3 (-1.1, 0.5) | 0.8 (0.8, 0.9) | 0.9 (0.8, 0.9) | -0.0 (-0.1, 0.0) | -0.3 (-0.9, 0.3) | |
| T4 | 0.8 (0.8, 0.8) | 0.8 (0.8, 0.9) | -0.0 (-0.1, 0.0) | -0.1 (-0.7, 0.6) | 0.8 (0.8, 0.8) | 0.8 (0.8, 0.9) | -0.0 (-0.1, 0.0) | -0.1 (-0.7, 0.5) | |
| * Adjusted for age, gender, educational level, and job facility.  **†** The number of participants at T2, T3 and T4 was 61 in the control group, and 50 in the intervention group.  SES, standardized effect sizes; PTSD, post-traumatic stress disorder; HR-QoL, health-related quality of life. | | | | | | | | |  |

**References**

World Health Organization. (‎2020)‎. *Doing what matters in times of stress: an illustrated guide*. World Health Organization. <https://apps.who.int/iris/handle/10665/331901>

World Health Organization. (‎2021)‎. *Self-Help Plus (SH+): a group-based stress managment course for adults*. World Health Organization. <https://www.who.int/publications/i/item/9789240035119>

World Health Organization. (2016). *Problem management plus (‎‎‎‎‎‎PM+)‎‎‎‎‎‎: individual psychological help for adults impaired by distress in communities exposed to adversity*. World Health Organization. <https://www.who.int/publications/i/item/WHO-MSD-MER-16.2>
